# Does Sphincteroplasty Predispose to Bile Duct Cancer?

**DOI:** 10.1155/1999/93568

**Published:** 1999-04

**Authors:** Russell W. Strong

**Affiliations:** Department of Surgery Princess Alexandra Hospital Ipswich Road, Woolloongabba Queensland 4102 Australia


### References

[1] Ginés, P., Arroyo, V., Vargas, V., Planas, R, Casafont, F., Panés, J., Hoyos, M., Viladomiu, L., Rimola, A., Morillas, R., Salmerón, J. M., Ginés, A., Esteban, R. and Rodés, J. (1991). Paracentesis with intravenous infusion of albumin as compared with peritoneovenous shunting in cirrhosis with refractory ascites. *New England Journal of Medicine*, **13**, 341–343.

[2] Arroyo, V., Ginés, P., Gerbes, A. L., Dudley, F. J., Gentilini, P., Laffi, G., Reynolds, T. F., Ring- Larsen, H. and Schölmerich, J. (1996). Definition and diagnostic criteria of refractory ascites and hepatorenal syndrome in cirrhosis. *Hepatology*, **23**, 164–176.

[3] Schrier, R. W., Arroyo, V., Bernardi, M., Epstein, M., Henriksen, J. H. and Rodés, J. (1988). Peripheral arteriolar vasodilatation hypothesis a proposal for the initiation of renal sodium and water retention in cirrhosis. *Hepatology*, **8**, 1151–1157.

[4] Barker, H. G. and Reemtsma, K. (1960). The portacaval shunt operation in patients with cirrhosis and ascites. *Surgery*, **48**, 142–154.

[5] Welch, H. F., Welch, C. S. and Carter, J. H. (1964). Prognosis after surgical treatment of ascites: Results of side-to-side shunt in 40 patients: *Surgery*, **56**, 75–82.

[6] Burchell, A. R., Rousselot, L. M. and Panke, W. F. (1968). A seven-year experience with side-to-side portacaval shunt for cirrhotic ascites. *Annals of Surgery*, **168**, 655–670.

[7] Franco, D., Vons, C., Traynor, O. and Smadja, C. (1988). Should portasystemic shunt be reconsidered in the treatment of intractable ascites in cirrhosis? *Archives of Surgery*, **123**, 987–991.

[8] Orloff, M. J., Orloff, M. S., Orloff, S. L. and Girard, B. (1997). Experimental, clinical and metabolic result of side-to-side portacaval shunt for intractable cirrhotic ascites. *Journal of the American College of Surgeons*, **184**, 557–569.

[9] Ochs, A., Rössle, M., Haag, K., Hauenstein, K. H., Deibert, P., Siegerstetter, V., Huonker, M., Langer, M. and Blum, H. (1995). The transjugular intrahepatic portasystemic stent-shunt procedure for refractory ascites. *New England Journal of Medecine*, **332**, 1192–1197.

[10] Lebree, D., Giuily, N., Hadengue, A., Vuilgrain, V., Moreau, R., Poynard, T., Gadano, A., Lassen, C., Benhamou, J. P. and Erlinger, S. (1996). Tansjugular intrahepatic portasystemic shunts comparison with paracentesis in patients with cirrhosis and refractory ascites: a randomized trial. *Journal of Hepatology*, **25**, 135–144.

[11] Guevara, M., Ginés, P., Fernández-Esparrach, G., Sort, P., Salmerón, J. M., Jiménez, W., Arroyo, V. and Rodés, J. (1998). Reversibility of hepatorenal syndrome by prolonged administration of ornipressin and plasma volume expansion. *Hepatology*, **27**, 35–41.

[12] Gonwa, T. A., Morris, C. A., Goldstein, R. M., Husberg, B. S. and Klintmalm, G. B. (1991). Long-term survival and renal function following liver transplantation in patients with and without hepatorenal syndrome. Experience in 3000 patients. *Transplantation*, **51**, 428–430.

Prof. J. Rodés, MD
Liver Unit
Hospital Clinic
University of Barcelona
Spain

### Does Sphincteroplasty Predispose to Bile Duct Cancer?

### ABSTRACT

*Hakamada, K., Sasaki, M., Endoh, M., Itoh, T., Morita, T. and Konn, M. (1998) Late development of bile duct cancer after sphincteroplasty: A ten- to twenty-two-year follow-up study. Surgery; 121, 488–492.*

*Background:* **Transduodenal sphincteroplasty is designed to destroy the sphincteric muscle fibers, producing a terminal choledochoduodenostomy. In the absence of Oddi's sphincter, intestinal contents with both activated pancreatic juice and bacterial flora are refluxed into the bile duct and remain there for a prolonged time. The long-term effect of producing the reflux has not been evaluated to date.**

*Methods:* **One hundred nineteen consecutive patients undergoing transduodenal sphincteroplasty between February 1973 and July 1984 were included in this study. Postoperative clinical courses of 108 patients could be evaluated by means of a retrospective review of the hospital records. Median follow-up was 18 years.**

*Results:* **Eight cases (7.4%) of primary bile duct cancer were found among the 108 cases at intervals of 1 to 20 years after sphincteroplasty. Two patients has concurrent hepatolithiasis. The patency of sphincteroplasty was confirmed in all cases, and the bile was infected in seven cases. Pathologic specimens obtained demonstrated cholangiocarcinomas and various degrees of atypical hyperplastic lesions under the background of chronic cholangitis.**

*Conclusions:* **Chronic cholangitis can be an important causative factor in late development of bile duct cancer after sphincteroplasty. Any patients treated with choledochoduodenostomy should be closely monitored for late cholangiocarcinoma. (*Surgery*, 1997; *121*: 488–492).**

*Keywords:* Bile duct carcinoma, sphincteroplasty, choledochoduodenostomy

### PAPER DISCUSSION

The report by Hakamada and colleagues is interesting, and on first perusal, raises a potentially disturbing message. They reviewed 119 consecutive patients on whom a transduodenal sphincteroplasty had been performed more than 10 years before and were able to evaluate 108 of the patients by means of a retrospective review of hospital records. They found eight patients who developed cholangiocarcinoma at intervals of one to 20 years following sphincteroplasty. Four of the patients were diagnosed less than 10 years and four during the second decade after sphincteroplasty. They confirmed the patency of the sphincteroplasty in all cases and, in addition to cholangiocarcioma, demonstrated various degrees of hyperplastic lesions and atypia of the biliary epithelium. They showed infected bile from samples obtained by direct puncture of the intrahepatic bile duct adjacent to the tumour in seven of the eight patients. They concluded that the absence of the sphincter mechanism allowed intestinal contents, with activated pancreatic juice and bacterial flora, to reflux into the biliary tree and that chronic cholangitis ensued and this was an important causative factor in the late development of bile duct cancer. At first site, this seems a plausible hypothesis.

Two of the patients would appear to have had little carcinogenic predisposition – acalculous cholecystitis and chronic pancreatitis and developed carcinoma 20 and 18 years respectively after surgery. One patient had gall bladder stones as well as choledocholithiasis while the others had choledocholithiasis with one diagnosed as hepatolithiasis. It is difficult for the reviewer to determine whether these cases were variants of hepatolithiasis or whether these were secondary stones. Cholangiocarcinoma is a well known complication of hepatolithiasis, even after apparent complete removal of all calculi [1].

The diagnosis of carcinoma was made one year after sphincteroplasty in one patient and it would have to be concluded that this was present at the time of operation, albeit undiagnosed. The two patients whose malignancy was diagnosed at five years, presumably had precancerous changes or carcinoma *in-situ* which may have been accelerated by the procedure. It would not be surprising that bile culture from obstructed ducts proximal to the carcinoma would be infected. This could well be effect, rather than cause, although it is conceded that culture of bile from the biliary tree is likely to contain bacteria after any biliary – enteric anastomosis.

There is some experimental evidence that would support the hypothesis that biliary – enteric anastomosis is a carcinogenic procedure [2, 3]. If transduodenal sphincteroplasty does make the patient susceptible to the development of cholangiocarcinoma, it would be expected that with all the choledochoduodenostomies and sphincteroplasties (the latter could be regarded as an internal choledocho-duodenostomy) performed in the past, there would be numerous reports of late cholangiocarcinoma. The reviewer is aware of only three reports of late cholangiocarcinoma following biliary – enteric anastomosis for benign disease [4 – 6] and has personal experience of three cases. It is possible that the condition has been under-reported. No increased risk of bile duct cancer was found in patients who underwent choledochoduodenostomy or sphincterotomy combined with cholecystectomy in the report by Ekbom *et al.* [7]. Nor was their any increased risk of cancer following sphincterotomy for stones in the common bile duct in a population based study from Sweden [8]. It could be argued that destruction of the Sphincter of Oddi by endoscopic sphincterotomy is less likely to lead to reflux of duodenal contents as does formal sphincteroplasty but this has not been tested.

Does sphincteroplasty or any other biliary-enteric anastomotic procedure predispose to bile duct cancer? There is some evidence to support the hypothesis but the jury is still out. The report by Hakamada may prompt review by other investigators and place the risk of developing cholangiocarcinoma after biliary-enteric anastomosis in its true perspective.

### *References*

[1] Chijiiwa, K., Ichimiya, H., Kuroki, S., Koga, A. and Nakayama, F. (1993). Late development of cholangiocarcinoma after treatment of hepatolithiasis. *Surg. Gynecol. Obstet.*, **177**, 279-282.

[2] Kurumado, K., Nagai, T., Kondo, Y. and Abe, H. (1994). Long term observations on morphological changes of choledochal epithelium after choledochoenterostomy in rats. *Dig. Dis. and Sciences*, **39**, 809-820.

[3] Tajima, Y., Eto, T., Tsunoda, T., Tomiaka, T., Inoue, K., Fukahori, T. and Kanematasu, T. (1994). Induction of extrahepatic biliary carcinoma by *N*-nitrosobis amine in hamsters given cholecystoduodenostomy with dissection of the common duct. *Jpn. J. Cancer Res.*, **85**, 780-788.

[4] Shields, H. (1977). Occurrence of an adenocarcinoma at the choledochoenteric anastomosis 14 years after pancreatoduodenectomy for benign disease. *Gastroenterol.*, **72**, 322-324.

[5] Haratake, J., Horie, T. and Takeda, N. (1983). Occurrence of intrapancreatic choledochal carcinoma 9 years after choledochojejunostomy for benign disease. *Jpn. J. Cancer Clin.*, **29**, 1367-1370.

[6] Herba, M., Casola, G., Bret, P., Lough, J. and Hampson, L. (1986). Cholangiocarcinoma as a late complication of choledochoenteric anastomoses. *AJR*, **147**, 513-515.

[7] Ekbom, A., Hsieh, C. and Yuen, J. (1993). Risk of extrahepatic bile duct cancer after cholecystectomy. *Lancet.*, **42**, 1262-1265.

[8] Karlson, B., Ekbom, A., Arvidsson, A., Yuen, J. and Krusema, U. (1997). Population based study of cancer risk and relative survival following sphincterotomy for stones in the common bile duct. *Br. J. Surg.*, **84**, 1235-1238.

Russell W Strong
Professor of Surgery
Department of Surgery
Princess Alexandra Hospital
Ipswich Road
Woolloongabba
Queensland
Australia 4102

